# Metabolomic Analysis Uncovers Lipid and Amino Acid Metabolism Disturbance During the Development of Ascites in Alcoholic Liver Disease

**DOI:** 10.3389/fmed.2022.815467

**Published:** 2022-06-13

**Authors:** Cheng Cheng, Ming-xi Zhou, Xian He, Yao Liu, Ying Huang, Ming Niu, Yi-xuan Liu, Yuan Gao, Ya-wen Lu, Xin-hua Song, Hui-fang Li, Xiao-he Xiao, Jia-bo Wang, Zhi-tao Ma

**Affiliations:** ^1^College of Chinese Medicine and Food Engineering, Shanxi University of Traditional Chinese Medicine, Jinzhong, China; ^2^Department of Hepatology, The Fifth Medical Center of Chinese PLA General Hospital, Beijing, China; ^3^School of Traditional Chinese Medicine, Capital Medical University, Beijing, China

**Keywords:** ascites, alcohol liver disease, untargeted metabolomic, lipid metabolism disturbance, amino acid metabolism disturbance

## Abstract

Ascites is one of the most common complications of cirrhosis, and there is a dearth of knowledge about ascites-related pathologic metabolism. In this study, 122 alcoholic liver disease (ALD) patients, including 49 cases without ascites, 18 cases with mild-ascites, and 55 cases with large-ascites (1) were established according to the International Ascites Club (2), and untargeted metabolomics coupled with pattern recognition approaches were performed to profile and extract metabolite signatures. A total of 553 metabolites were uniquely discovered in patients with ascites, of which 136 metabolites had been annotated in the human metabolome database. Principal component analysis (PCA) analysis was used to further identify 21 ascites-related fingerprints. The eigenmetabolite calculated by reducing the dimensions of the 21 metabolites could be used to effectively identify those ALD patients with or without ascites. The eigenmetabolite showed a decreasing trend during ascites production and accumulation and was negatively related to the disease progress. These metabolic fingerprints mainly belong to the metabolites in lipid metabolism and the amino acid pathway. The results imply that lipid and amino acid metabolism disturbance may play a critical role in the development of ascites in ALD patients and could be a potent prognosis marker.

## Introduction

Alcoholic liver disease (ALD), caused by excessive alcohol consumption, is a global healthcare problem with enormous social, economic, and clinical consequences (3–6). Alcoholic cirrhosis is an advanced type of alcohol-related liver disease, resulting from the death of functional hepatocytes and their replacement with scar tissue. Alcoholic cirrhosis (ALC) develops in 10–20% of chronic heavy drinkers after several years of heavy drinking (7, 8). Ascites is the most common complication of cirrhosis (9, 10). It is the pathologic accumulation of fluid in the peritoneum caused by portal hypertension. Over a 10-year follow-up period, 50% of patients with previously compensated cirrhosis are expected to develop ascites (11). As a marker of hepatic decompensation, ascites is associated with a poor prognosis, with only a 56% survival rate of 3 years after onset (12). In addition, they serve as a warning indication concerning liver failure. Morbidity is also increased due to the risk of additional complications, such as spontaneous bacterial peritonitis and hepatorenal syndrome. Clinically, the prognosis of ascites largely depends on the underlying cause (i.e., the primary disease). To date, there is still a lack of efficient tools for improving ascites, yet further understanding of the pathophysiology of ascites is crucial.

Through quantitative analysis of metabolites, metabolomics is a useful approach for the study of pathological metabolites during disease progress, as well as the search for related metabolic biomarkers and potent therapeutic targets. Non-targeted metabolomics has been applied in ALD-related metabolite research and found energy supply disturbance as an underlying mechanism of the development of ALD (13). However, there is still little known regarding the metabolites associated with the development and progression of ascites. The objective of this study is to characterize the sera metabolite fingerprints in ALD patients with and without ascites to provide cues for the treatment or prognosis of clinical ascites.

## Materials and Methods

### Study Cohort

Patients with ALD who were seen at The Fifth Medical Center of Chinese PLA General Hospital between 1 January 2015 and 31 December 2017 were screened in the study. The enrolled patients were diagnosed according to ALD guidelines (4) and liver cirrhosis guidelines (1). Patients with other etiologies of chronic liver diseases such as viral hepatitis, cholestatic liver diseases, and autoimmune liver diseases were excluded. ALD patients with ascites that were not infected and not associated with the development of the hepatorenal syndrome were included. Baseline demographic and clinical data were obtained. Serum samples were stored at −80°C until the analysis. In the end, a total of 122 patients constituted the study cohort: 49 ALD patients without ascites (NA), 18 ALD cases with mild ascites, and 55 ALD cases with large ascites.

Written, informed consent was obtained from all participants. The study was approved by the Ethics Committees of The Fifth Medical Center of Chinese PLA General Hospital, Beijing.

### Preparation of Serum Sample

According to medical literature (14), the biobank serum was processed. Quality control (QC) samples were prepared by mixing each sample with 10 μl to be analyzed. Chromatographic conditions and mass spectrometry conditions were processed according to the literature (13).

### Reagent and Equipment

Agilent Technologies Liquid Chromatograph (Model: 1290 Infinity, United States), Agilent Technologies Quadrupole Time-of-Flight Mass Spectrometer: Agilent Technologies Q-TOF LC/S (Model: 6550 iFunnel), Ultra-low temperature refrigerator (Model: DW-86L 728J, Haier Group), Vortex mixer (Model: Vortex Genie 2, Scientific Industries, United States), table top high speed refrigerated centrifuge (Model: TGL-16M, Shandong Boke Scientific Instrument Co., Ltd., Jinan, China), vacuum centrifugal concentrator (Model: CV200, Beijing Jiaimu Technology Co., Ltd., Beijing, China), automatic snowflake ice maker (Model: IMS-100, Changshu Xueke Electric Appliance Co., Ltd., Changshu, China), and Hisense refrigerator (Model: BCD-206H, Hisense Group Co., Ltd., Beijing, China).

Methanol (Batch Number: 196063, Chromatography pure, Fisher Chemical, Shanghai, China), Acetonitrile (Batch Number: 201643, Chromatography pure, Fisher Chemical, United States), Formic acid (Batch Number: 171662, Chromatographic purity, Fisher Chemical, Shanghai, China), and Wahaha Purified Drinking Water (Hangzhou Wahaha Group Co., Ltd., Shanghai, China).

### Data Processing and Statistical Analysis

Data processing and statistical analysis were carried out based on those by Huang et al. (13–15). Briefly, metabolomic data were normalized by the inclusion of multiple internal standards and pool calibration–response correction in MetaboAnalyst version 4.0 after being processed in MassHunter Profinder^1^. The normalized data were analyzed using the Mann–Whitney *U* test with *p* < 0.05 set as the level of statistical significance. These variables were identified in the human metabolome database. Descriptive statistics for continuous variables were presented as the mean, with SD for normally distributed parameters, or median, with corresponding upper and lower quartiles for non-normally distributed parameters. For categorical data, numbers and percentages were used. Appropriate comparison tests including the chi-square test, analysis of variance, and Mann–Whitney *U* test were used for comparison among groups for categorical and continuous variables. The significance level for all statistical tests was set at 0.05, and adjusted *p* values < 0.05 in multiple comparisons. All statistical analyses were performed using SPSS 25 software (IBM, Chicago, IL, United States). Principal component analysis (PCA) and orthogonal partial least squares discrimination analysis (OPLS-DA) were performed in SIMCA-P 14.1 software (Paris, France). The Nightingale rose diagram was created with Anaconda-Navigator.

### Metabolomic Analysis

We performed several comparisons to find the metabolites with significant differential expressions among groups (*p* < 0.05): (1) comparison between those with mild ascites and those without ascites (MA/NA); and (2) comparison between those with large ascites and those without ascites (LA/NA). We used the following two approaches to explore the metabolites associated with disease progression: first, the shared metabolites of the two comparisons (MA/NA and LA/NA) were identified; these were the metabolites uniquely related to ALD independence of ascites states; next, we characterized metabolites that were identified in MA + LA/NA metabolites; these represented metabolites associated with ascites in patients with ALD. We then annotated these selected metabolites according to the Human Metabolome Database. Based on those annotated metabolites, hierarchical clustering was used to develop a metabolic fingerprint consisting of a cluster of metabolites using the area under the curve (AUC) and *p* values in differentiating ALD patients with different ascites states, from NA to MA and LA (16, 17). We used the threshold combination of *p* < 0.05 “VIP ≥ 1” to screen the differences. To carry out more rigorous screening, we sorted log FC values within the range of *p* < 0.05 “VIP ≥ 1” (log FC values < 1 are more meaningful; log FC > −1, the smaller the more meaningful). The ascites-associated fingerprint metabolites were then projected to the eigenmetabolite by dimension reduction to visually observe the difference between groups from a series of fingerprint metabolites as previously described. Finally, the alterations of the metabolic fingerprint between groups were displayed with the Nightingale rose diagram using the normalized relative intensity value of each metabolite within different groups.

## Results

### Baseline Demographics and Clinical Characteristics of the Study Cohort

The detailed baseline demographic and clinical characteristics of patient groups with NA, MA, and LA are shown in Tables 1, 2. As can be seen in the Table 2, most patients were male (*n* = 120, 98.36%), with the exception of two who were female. No significant differences were seen in age or body mass index (BMI) among the three groups. The drinking duration for the patients was greater than 20 years, and the estimated daily alcohol consumption amounts were approximately 196 g, 224 g, and 240 g in the NA, MA, and LA groups, respectively. At the time of enrollment, the percentage of patients experiencing cirrhosis was 69.39% in the NA group and 100% in the MA and LA groups. Serum ALT, AST, ALP, GGT, and creatinine (CRE) exhibited no significant differences among the three groups. Total bilirubin (TBil), direct bilirubin (DBil), and total bile acid (TBA) were increased significantly in both the MA and LA patients, while serum albumin (ALB), cholinesterase (CHE), total cholesterol (TC), and triglycerides (TG) gradually decreased. The levels of prothrombin time (PT) and international normalized ratio (INR) in the MA and LA patients were higher than that of patients in the NA group. The white blood cell counts and platelets (PLT) in the MA and LA groups were both less than that of the NA patients. Patients in the MA and LA groups had a significantly higher MELD score, suggesting increased severity of liver dysfunction and increased mortality by 3 months. These results suggest a disturbance of liver function during ascites advancement.

**TABLE 1:** Diagnostic criteria and definitions of ALD with or without ascites (1).

Definitions and diagnostic criteria	Descriptions
ALD	Consists of three parts: (1) patients with a history of excessive alcohol consumption of >20 g/day in females and >40 g/day in males over 5 years; (2) patients with liver injury by clinical manifestation, abnormal liver biochemistries, radiographic imaging, and/or histological findings; and (3) other causes of liver diseases (excluded)
Ascites	A condition in which the liver is scarred and permanently damaged based on relevant ultrasound or histological findings
Mild ALD ascites	Patients with ascites is only detectable by an examination such as ultrasound, without any complications of advanced liver disease
Large ALD ascites	Consist of two parts: (1) patients with ascites are detectable by an examination such as ultrasound and (2) patients with ascites presented with moderate symmetrical distension of the abdomen and marked abdominal distension
Non-ascites	Patients diagnosed with ALD but without the condition of ascites in terms of ultrasound or histological findings

**TABLE 2:** Comparison of characteristics of NA, MA, and LA patients.

	NA (*n* = 49)	MA (*n* = 18)	LA (*n* = 55)	*P* value
Age/years	49 ± 7	51 ± 6	51 ± 9	0.465
Male/n%	49 (100.00)	18 (100.00)	53 (96.36)	–
BMI, kg m^–2^	24.42 (21.92, 27.36)	22.77 (21.38, 25.21)	23.82 (21.50, 27.75)	0.432
ALT, U L^–1^	30.00 (19.50, 40.00)	27.00 (21.00, 34.75)	26.00 (15.00, 38.00)	0.208
AST, U L^–1^	40.00 (25.00, 63.00)	49.50 (35.50, 80.25)	47.00 (29.00, 79.00)	0.271
AST/ALT	1.5 ± 0.9	1.9 ± 0.7	2.1 ± 0.7	0.000
ALP, U L^–1^	123.00 (91.00, 174.50)	148.00 (89.00, 218.00)	142.00 (102.00, 166.00)	0.396
GGT, U L^–1^	60.00 (33.00, 130.50)	94.5 (36.50, 269.50)	56.0 (38.00, 169.00)	0.681
TBil, μmol L^–1^	23.50 (14.55, 39.90)	37.90 (24.93, 58.55)	45.90 (25.90, 83.50)	0.002
DBil, μmol L^–1^	9.80 (5.70, 23.15)	20.05 (11.50, 34.55)	27.00 (13.00, 57.10)	0.001
TBA, μmol L^–1^	17.00 (9.50, 84.50)	68.50 (39.75, 99.75)	61.00 (30.00, 114.00)	0.017
ALB, g L^–1^	35.00 (28.00, 39.50)	29.50 (26.75, 33.25)	27.00 (25.00, 32.00)	0.000
CHE, U L^–1^	4742 (3213, 5966)	2850 (2242, 3821)	2426 (1737, 3320)	0.000
CRE, μmol L^–1^	72.00 (62.50, 78.00)	66.00 (64.00, 72.50)	75.00 (62.00, 91.00)	0.271
PT, seconds	12.00 (10.95, 14.70)	13.65 (11.90, 15.88)	14.90 (12.50, 16.50)	0.001
INR, IU	1.04 (0.96, 1.29)	1.23 (1.04, 1.58)	1.28 (1.09, 1.45)	0.001
TC, mmol L^–1^	3.83 (3.21, 5.16)	3.01 (2.56, 4.77)	3.12 (2.65, 3.84)	0.008
TG, mmol L^–1^	1.09 (0.86, 1.56)	0.87 (0.64, 1.11)	0.92 (0.59, 1.19)	0.011
WBC, mm^3^	5570 (3715, 48800)	4690 (2210, 7793)	4350 (2600, 6990)	0.006
PLT, 10^9^⋅L^–1^	110 (73, 202)	65 (46, 113)	86 (51,122)	0.033
With HE, %	1 (2.04)	1 (5.56)	2 (3.64)	–
With cirrhosis, %	34 (69.39)	18 (100.00)	55 (100.00)	–
Duration of drinking, years	20 (15, 30)	20 (20, 30)	20 (20, 30)	0.065
Estimated daily alcohol intake, g	196 (126, 280)	224 (140, 315)	240 (140, 280)	0.564
MELD score	8.96 (6.54, 12.47)	12.11 (9.38, 15.53)	14.35 (10.45, 17.43)	0.000

*Data are reported as mean ± SDs or median (upper quartile, lower quartile) unless otherwise noted as n (%). P values indicate the significant difference among the three groups analyzed by ANOVA. ALP, alkaline phosphatase; ALT, alanine aminotransferase; BMI, body mass index; CRE, creatinine; DBil, direct bilirubin; GGT, gamma-glutamyl transpeptidase; HE, hepatic encephalopathy; PLT, platelets; PT, prothrombin time; INR, international normalized ratio; TBA, total bile acid; TC, total cholesterol; TG, triglyceride; WBC, white blood cell.*

### Serum Metabolites Profiles in Alcoholic Liver Disease Patients With Ascites

**FIGURE 1:**
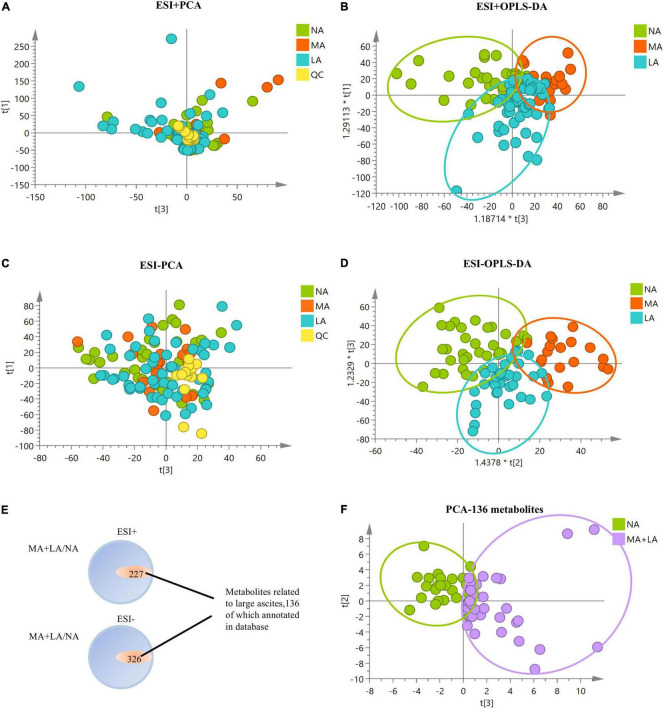
Metabolomics analysis of ALD patients with/without ascites. **(A–D)** PCA and orthogonal partial least squares discrimination analysis (OPLS-DA) plots with all metabolic variables in NA, MA, and LA patients under positive and negative models of mass spectrometry, respectively. **(E)** Metabolites with significant differences in MA and LA, in comparison to NA. There are 553 metabolites uniquely associated with ascites, with 136 metabolites annotated in the Human Metabolome Database. **(F)** PCA plot of MA + LA vs. NA with 136 annotated metabolites related to ascites. The QC samples are clustered together in the PCA plots, indicating stability and technical reproducibility.

To determine the metabolomic profiles associated with ascites in ALD, sera metabolites analysis of ALD patients with/without ascites was conducted, and significant alterations in the metabolomics profile in ALD patients with ascites from either positive or negative modes of mass spectrometry were observed (Figures 1A–D). The potential serum metabolites with significant differences (*p* < 0.05) in the MA and LA patients, compared to that of the NA patients, were obtained. It was shown that a total of 553 metabolites (including positive and negative patterns) were uniquely associated in patients with ascites, among which 136 metabolites were annotated in the Human Metabolome Database. Notably, these 136 metabolites effectively distinguished patients with/without ascites in the PCA model, indicating that these metabolites might have a close relationship with the ascites development in ALD-related cirrhosis patients (Figures 1E,F).

### Selection of Metabolic Fingerprints for a Unique Expression of Ascites in Alcoholic Liver Disease Patients

**FIGURE 2:**
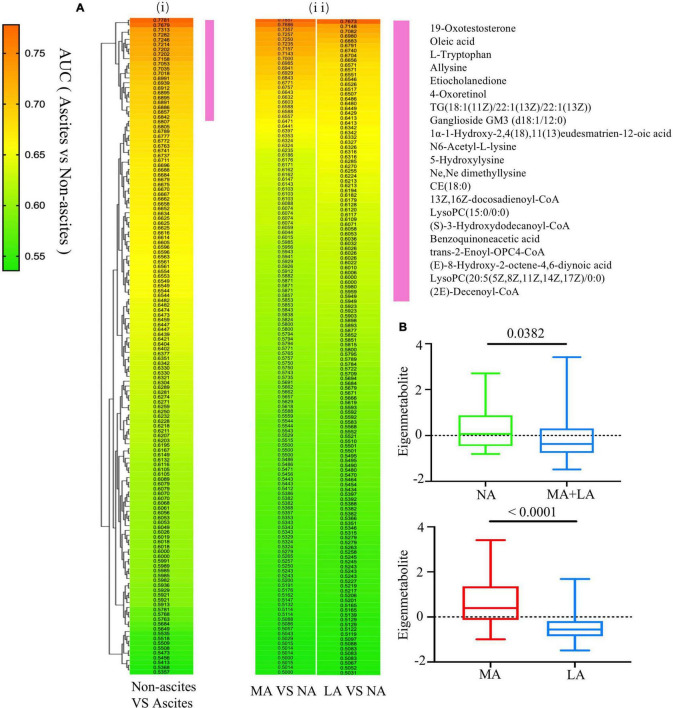
Selection of metabolic fingerprints uniquely expressed in ALD patients with ascites. **(Ai)** Hierarchical cluster analysis of the AUCs and *p* value assessing the discriminating accuracy of each of the 136 metabolites in differentiating ALD with or without ascites. **(Aii)** The corresponding metabolites’ AUC and *P* values in accessing MA or LA relative to NA. The vertical violet bar identifies the 21-metabolite clusters highly associated with ascites and the ascites-associated metabolite fingerprints. **(B)** The decreasing trend of the eigenmetabolite (21 fingerprint metabolites) in ALD patients without ascites and different stages of ascites.

The AUC and *p* values of the 136 annotated metabolites related to ascites were calculated, respectively, to identify the unique metabolomic fingerprints with a distinguished expression between patients with (MA and LA groups) or without (NA group) ascites. The values obtained were used for hierarchical cluster analysis and displayed in a heat map. It was shown that the highest correlation cluster with the top 21 metabolites was highly related to the ascites progress (Figure 2Ai). The AUC for these metabolites clusters ranged from 0.6842 to 0.7781, with *P* values < 0.05 (Supplementary Table 1). This cluster was also effective in distinguishing between the MA and NA groups, as well as the LA and NA groups (Figure 2Aii). Next, we calculated the eigenmetabolite of these 21 metabolites by reducing the dimensions and observed a significant decrease in the eigenmetabolite from the NA to MA + LA patients (Figure 2B). In addition, a significant decrease in the eigenmetabolite was also found from the MA to LA patients (Figure 2B). Hence the eigenmetabolite was negatively correlated with the ascites clinical-stage progress.

### Use of Metabolic Fingerprints to Unravel Underlying Pathophysiological Changes When Ascites Occur

**FIGURE 3:**
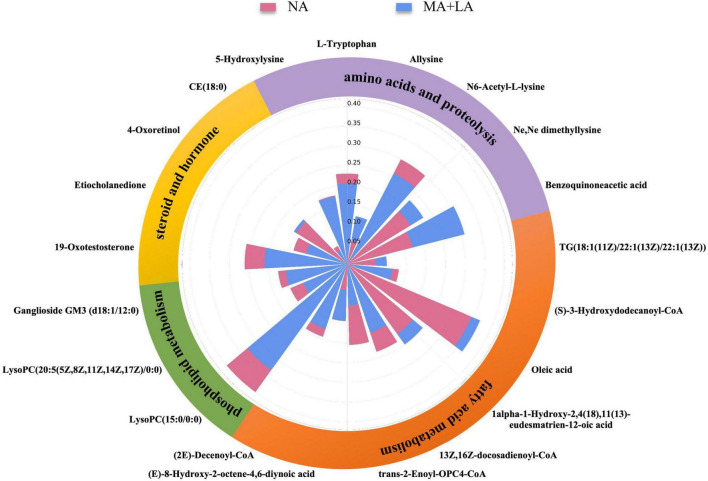
Use of metabolic fingerprints to unravel underlying pathophysiological changes during the progression of ascites. The Nightingale rose diagram of ascites-related metabolic fingerprints. The value of the relative intensity of each metabolite was normalized within the two groups. The whole set of metabolites in the fingerprint was classified into different metabolic pathways, including fatty acid metabolism, phospholipid metabolism, steroids and hormones, amino acids, and proteolysis.

**FIGURE 4:**
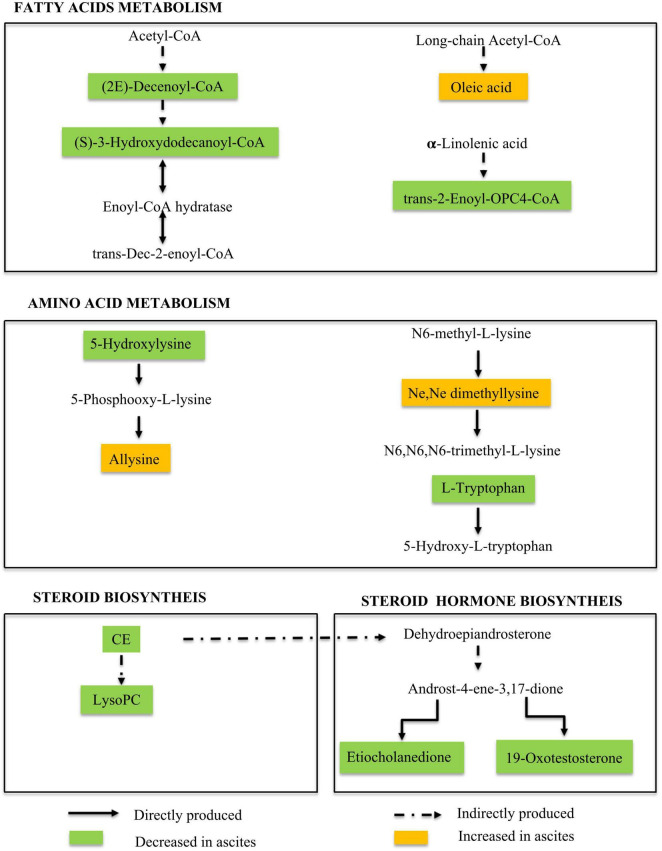
Metabolic pathways involved in ALD patients with ascites. Our data suggested the dysregulation in the fatty acid metabolism pathway, amino acid metabolism pathway, and steroid and hormone biosynthesis pathway during ALD-related ascites progression.

To better understand the role of these metabolites in the pathological changes in patients with ascites, a Nightingale rose diagram of the distinguished expressed metabolites in ALC patients with ascites was created (Figure 3). The related pathways were also detected and are shown in Figure 4.

### Ascites-Related Metabolic Fingerprints Show Metabolites Related to Lipid Metabolism Pathways

The results showed that the 21 ascites-related metabolic fingerprints contained 14 metabolites associated with lipid metabolism pathways (Figure 3 and Supplementary Table 2). Metabolites associated with fatty acids, phospholipids, steroids, and the hormone metabolism pathway were found in ALD patients with ascites.

There are eight fatty acid related fingerprints of ALD patients with ascites, and four fingerprints [oleic acid: 1α-1-hydroxy- 2,4(18),11(13)-eudesmatrien-12-oic acid; (E)-8-hydroxy-2-octene-4,6-diynoic acid; TG(18:1 (11Z)/ 22:1(13Z)/22:1(13Z))] were upregulated in the MA and LA groups, while the other four fingerprints [(S)-3-hydroxydodecanoyl-CoA; 13Z,16Z-docosadienoyl-CoA; (2E)-decenoyl-CoA; *trans*-2-enoyl-OPC4- CoA] were downregulated.

In addition, 3 phospholipid-related metabolites were contained in the 21 fingerprints in ALD patients with ascites. They were LysoPC(15:0/0:0), LysoPC (20:5(5Z,8Z,11Z, 14Z,17Z)/0:0), and ganglioside GM3 (d18:1/12:0) and were all downregulated in the MA + LA groups.

In addition, 4 steroids and hormone-related metabolites were contained in the 21 fingerprints in ALD patients with ascites. In the steroids, 4-oxoretinol was upregulated, while CE (18:0) was downregulated in the MA and LA groups. The two hormones (19-oxotestosterone; etiocholanedione) were both significantly decreased in the MA and LA groups (Figure 3 and Supplementary Table 2).

### Ascites-Related Metabolic Fingerprints Show Metabolites Related to Amino Acid Metabolism Pathways

The alternation of amino acid-related metabolites was also detected during ascites production and accumulation in ALD patients and 6 amino acid metabolites were contained in the 21 ascites-related metabolic fingerprints. It was observed that the metabolites, L-tryptophan, N6-acetyl-L-lysine, and 5-hydroxylysine were decreased in ALD patients with ascites. Conversely, benzoquinone acetic acid (BQA), allysine, and Ne,Ne dimethyllysine were elevated (Figure 3 and Supplementary Table 2).

## Discussion

In this study, we performed non-targeted full-spectrum sera metabolomics to analyze metabolism alternation in ALD patients with or without ascites. The results showed that, compared to ALD patients without ascites, a total of 553 metabolites were uniquely expressed in patients with ascites, of which 136 metabolites were annotated in the human metabolome database. These 136 metabolites distinguished patients with/without ascites in the PCA model. Moreover, 21 ascites-related fingerprints were further separated through the PCA analysis. These fingerprints were mainly intermediate metabolites in lipid and amino acid metabolism pathways. Our results showed that the metabolic products of the fatty acid, steroid, and phospholipid metabolism pathways in ALD patients with ascites were disordered. It is well known that long-term and excessive alcohol consumption inhibits fatty acid oxidation, causing a significant shift in cellular energy supply from fatty acid oxidation to fatty acid synthesis (18). In addition, excessive alcohol consumption enhances acetaldehyde production and accumulation. Acetaldehydes not only disrupt the hepatocellular structure but also cause a disturbance in intrahepatic lipid metabolism and result in increased free fatty acids in serum, which were observed in our previous studies on the metabolic fingerprint in patients with chronic alcoholic liver disease without cirrhosis, compared to normal persons (13). To note, compared to the non-ascites ALD patients, the fatty acid, steroid, and phospholipid metabolism pathways were disordered in ALD patients with ascites. This implies that continual disruption in lipid metabolism is characteristic of ALD exacerbation.

Hepatic fatty acid metabolism is tightly regulated by multiple interrelated transcriptional and signaling pathways in the normal liver. Elevated TG(18:1(11Z)/22:1(13Z)/22:1(13Z)) in the MA and LA groups could be convincing evidence of fatty acid metabolism disorder in ALD patients with ascites. Another fatty acid-related metabolite, oleic acid was also upregulated in ALD patients with ascites. Oleic acid has been reported to cause toxic damage to rat hepatocyte cells and activate MAPK/TLR4 to induce lipid storage (19). Oleic acid could also induce steatosis in HepG2 cells in a dose-dependent manner, decrease the expression of PPAR-α and superoxide dismutase-1, increase lipid peroxide production and lead to cell proliferation inhibition (20). These results imply that oleic acid upregulation might contribute to ascites formation and accumulation in ALD patients through inducing lipid metabolism disorder, hepatocyte death, and oxidative stress. It might be a potent therapeutic target for ALD and ascites treatment.

Alterations in the level of CoQ-related fatty acid metabolites, such as (S)-3-hydroxydodecanoyl-CoA, 13Z,16Z-docosadienoyl-CoA, (2E)-decenoyl-CoA, and *trans-*2-enoyl-OPC4-CoA were also detected in ALD patients with ascites. CoQ is the mitochondrial respiratory chain carrier and a potent membrane anti-oxidant (13, 21, 22). CoQ and a depletion in the related metabolites would in turn disturb the triacylglycerol biosynthesis pathway and lead to disorder in energy metabolism during ascites progress. It has been reported that docosadienoyl-CoA participates in a number of enzymatic reactions and is always involved in the metabolic disorder known as *de novo* triacylglycerol biosynthesis pathway (23). 13Z,16Z-docosadienoyl-CoA, a component of docosadienoyl-CoA, was downregulated in ALD patients with ascites, which implies that triacylglycerol biosynthesis pathway disorder might be a critical contributor to ascites. In contrast, alcohol metabolism triggers intracellular oxidative stress (13, 24, 25). As an antioxidant, the reduction of CoQ and the related metabolites in ALD patients may enhance oxidative stress and worsen the liver injury, which might further contribute to ascites production and accumulation in ALD patients.

Our results demonstrated that *trans-*2-enoyl-OPC4-CoA was downregulated during ascites production and progress. Interestingly, *trans-*2-enoyl-OPC4-CoA was also found to be significantly reduced in autoimmune hepatitis-related cirrhosis (AIH) as shown in our previous study (15). The similar liver response triggered by AIH compared with alcohol consumption suggests that *trans-*2-enoyl-OPC4-CoA downregulation might be a common characteristic of cirrhosis progress. This requires further study. Other fatty acid intermediate metabolites upregulated during the ascites process included (E)-8-hydroxy-2-octene-4,6-diynoic acid and 1α-1-hydroxy-2,4(18),11(13)-eudesmatrien-12-oic acid, but little is known in terms of their functions.

Phospholipids are the critical components of cell membranes and are essential for many functions, such as immune signal recognition and transcellular signal transduction (26). The signal originating from cell membranes may activate a signal cascade concerning cellular metabolism and thus cell fate, such as endoplasmic reticulum stress and mitochondrial function disruption (27). It has been reported that abnormal phospholipid levels are related to alcoholic liver cirrhosis. Our results further confirmed the phenomena and also showed the abnormal metabolism of phospholipids in ALD-related ascites patients. Gangliosides (GM3), one of the downregulated phospholipid fingerprints in ALD-related ascites patients, have various fatty acid compositions, including long-chain (LCFA) and ultra-long-chain (VLCFA) (28). It has been reported that VLCFA-GM3 is an agonist of the TLR4 signal pathway and plays an important role in the pathogenesis of metabolic disorders through TLR4-mediated innate immune signals (29, 30). The fact that GM3 experiences downregulation in ALD-related ascites patients presents a risk factor for ALD progression through mediating immune signals. In addition, 15:0 and 20:5 LysoPC, which act as detergents, were found downregulated in ALD patients with ascites.

Steroids always function as molecular signals (31). Steroid-related fingerprints, 4-oxoretinol, and CE (18:0) were found in ALD-related ascites patients. It has been reported that 4-oxoretinol could induce cell growth arrest and participate in the differentiation process of granulocytes (32). The increasing serum concentration of 4-oxoretinol during the ALD-related ascites progression suggests that the cell cycle of hepatocytes is arrested to inhibit liver regeneration. The hepatocytes injured by alcohol metabolism could not be replaced by hepatocyte proliferation to reconstitute liver function, which may further promote ALD progress as well as ascites formation and accumulation. CE (18:0) is a cholesterol fatty acid ester or simply a cholesterol ester (CE). It is much less polar than free cholesterol and appears to be the preferred form for transport in plasma and for storage (33, 34). CE (18:0) downregulation implies the disordered transportation and storage of steroids.

The hormones, 19-oxotestosterone and etiocholanedione, are also downregulated in ALD patients with ascites, which is in accordance with previous reports (25). In addition, etiocholanedione is an androgen catabolism 5-β metabolite, and its production has been reported to consume NAD^+^ and transfer NAD^+^ into NADH. The imbalance of NAD^+^/NADH has been a critical step in the energy metabolism disorder induced by massive alcohol metabolism (13, 15). The etiocholanedione production during ascites production and accumulation in ALD patients further enhances the consumption of NAD^+^, promoting ALD progress.

The disordered metabolism of amino acids *in vivo* was observed after feeding alcohol (35) and further confirmed in our results. The intermediate metabolites of amino acids, L-tryptophan, N6-acetyl-L-lysine, and 5-hydroxylysine decreased in ALD patients with ascites. Conversely, benzoquinone acetic acid, allysine, and Ne,Ne dimethyllysine increased. The disordered amino acid metabolism implies unregulated protein production in ALD patients with ascites, which will function pathologically.

Allysine, N6-acetyl-L-lysine, and Ne,Ne dimethyllysine are derivatives of lysine. iTRAQ-based quantitative proteomic analysis indicated that amino acid metabolism is implicated in the formation of HCC malignant ascites (36). Our results showed that the metabolic products of the amino acid metabolism pathway in ALD patients with ascites were disordered. Transcriptome analysis and gene identification work together to regulate pulmonary artery remodeling and demonstrate vascular smooth cell proliferation and inflammation. Pulmonary arterial remodeling presents a key step in the development of ascites syndrome (AS) (37). The extracellular matrix of collagen and elastin plays an important role in vascular elasticity (compliance) and integrity (38). Among the metabolites screened by us, allysine is used in the production of elastin and collagen and is essential in the crosslink formation that stabilizes collagen and elastin. It has been reported that tissue fibrogenesis results in the formation of allysine (39). This means that the upregulation of allysine in ALD patients with ascites might be characteristic of the enhanced liver fibrotic process and lead to scar formation and liver stiffening. In addition, it has been reported that the formation of allysine was positive relative to the severity of the myopathy (40). As the most abundant protein carbonyl in biological systems, the carbonylation of proteins involves an irreversible modification of essential amino acids (41). It enhances the pathogenesis of gastrointestinal disorders (42), interferes with the absorption of other amino acids in the intestinal tract, and affects the synthesis and utilization of protein, contributing to malnutrition and muscle atrophy in ALD patients. The urinary excretion of hydroxyproline and hydroxylysine was increased in proportion to the severity of the liver disease. In addition, urinary hydroxylysine excretion was suggested to be a critical index of hepatic collagen metabolism in chronic liver disease (43). Our results certified the points above. Moreover, serum lysine-related metabolism is not only related to liver fibrosis but also critical for the ascites progress. Benzoquinone acetic acid (BQA) is an oxidized form of homogentisic acid, which is decomposed by tyrosine. Homogentisic acid and BQA are usually excreted in the urine. The high level of BQA in ALD patients with ascites might be the result of portal hypertension. Additionally, the plasma tryptophan levels in cirrhosis patients are higher than in normal controls (44). However, the serum concentration of tryptophan in ALD patients with ascites is lower, compared to that of ALD patients without ascites. This difference may be due to serum tryptophan being kept in ascites, but this requires further research.

In this study cohort, there were only two females, accounting for 2.32% of the total ALD patients. The results are consistent with a retrospective analysis of ALD patients in China (45). This may be because more men in China consume alcohol, resulting in fewer female ALD patient samples for this study (46). We will continue to collect female ALD patient samples to explore their metabolomic fingerprints, and the relative metabolomic fingerprint differences between male and female ALD patients, in our future studies.

In summary, we found that ALD-related ascites is closely associated with amino acid and lipid metabolism disorders through non-targeted full-spectrum metabolomics analysis in ALD patients with/without ascites. These metabolic characteristics provide a new perspective for understanding the mechanism of ALD with ascites. Exploration of these metabolites as biomarkers or potential therapeutic targets in ALD patients with ascites is meaningful to future ascites medication. However, further research is still needed.

## Data Availability Statement

The original contributions presented in the study are included in the article/Supplementary Material, further inquiries can be directed to the corresponding authors.

## Ethics Statement

The studies involving human participants were reviewed and approved by the Ethics Committees of The Fifth Medical Center of Chinese PLA General Hospital. The patients/participants provided their written informed consent to participate in this study.

## Author Contributions

J-BW and Z-TM were responsible for the study concept and design. CC, M-XZ, XH, YL, YG, Y-XL, and X-HS performed the sample collection and conducted LC–MS data analysis. CC wrote the manuscript. MN and YH performed most of the experiments and data analysis. J-BW and CC provided assistance with investigation and writing the manuscript. CC, J-BW, Z-TM, and H-FL wrote and revised the manuscript. All authors contributed to the article and approved the submitted version.

## Conflict of Interest

The authors declare that the research was conducted in the absence of any commercial or financial relationships that could be construed as a potential conflict of interest.

## Publisher’s Note

All claims expressed in this article are solely those of the authors and do not necessarily represent those of their affiliated organizations, or those of the publisher, the editors and the reviewers. Any product that may be evaluated in this article, or claim that may be made by its manufacturer, is not guaranteed or endorsed by the publisher.

## Footnotes

1
https://www.metaboan-alyst.ca
