# Supplementary material for: Metabolomic Analysis Uncovers Lipid and Amino Acid Metabolism Disturbance During the Development of Ascites in Alcoholic Liver Disease
Source: Front Med (Lausanne). 2022 Jun 13;9:815467. doi: 10.3389/fmed.2022.815467 (PMC9234647; doi:10.3389/fmed.2022.815467)
Supplement: Supplementary file 1 [file Data_Sheet_1.docx]

**Table S1: Detailed information on 21 metabolites in the metabolic fingerprint of alcoholic liver disease** **with ascites.**

| **No.** | **Mode** | **MZ** | **RT** | **Name** | **HMDB** | **Formula** | **MA+LAvs NA** | | | |
| --- | --- | --- | --- | --- | --- | --- | --- | --- | --- | --- |
|  |  |  |  |  |  |  | **AUC** | **FC** | **log2(FC)** | **P** |
| 1 | ESI- | 281.2485 | 18.89min | Oleic acid | HMDB0000207 | C18H34O2 | 0.7679 | 1.0485 | 0.068338 | 0.69824 |
| 2 | ESI- | 165.0199 | 4.85min | Benzoquinoneacetic acid | HMDB0002334 | C8H6O4 | 0.6891 | 1.428 | 0.514 | 0.013243 |
| 3 | ESI- | 203.0821 | 2.45min | L-Tryptophan | HMDB0000929 | C11H12N2O2 | 0.7313 | 0.8927 | -0.16375 | 0.4383 |
| 4 | ESI- | 149.0276 | 18.60min | (E)-8-Hydroxy-2-octene-4,6-diynoic acid | HMDB0031515 | C8H6O3 | 0.6857 | 2.2281 | 1.1558 | 0.0016106 |
| 5 | ESI- | 144.0668 | 1.12min | Allysine | HMDB0001263 | C6H11NO3 | 0.7214 | 2.2152 | 1.1474 | 0.0037277 |
| 6 | ESI+ | 189.1265 | 13.23min | N6-Acetyl-L-lysine | HMDB0000206 | C8H16N2O3 | 0.7035 | 0.92323 | -0.11523 | 0.4551 |
| 7 | ESI+ | 163.1115 | 11.98min | 5-Hydroxylysine | HMDB0000450 | C11H14O | 0.7018 | 0.95455 | -0.067106 | 0.71844 |
| 8 | ESI+ | 175.1478 | 8.25min | Ne,Ne dimethyllysine | HMDB0013287 | C8H18N2O2 | 0.6991 | 1.0388 | 0.054911 | 0.82144 |
| 9 | ESI+ | 966.2686 | 11.58min | (S)-3-Hydroxydodecanoyl-CoA | HMDB0003936 | C33H58N7O18P3S | 0.6895 | 0.53788 | -0.89465 | 0.011864 |
| 10 | ESI+ | 920.2093 | 9.97min | (2E)-Decenoyl-CoA | HMDB0003948 | C31H52N7O17P3S | 0.6833 | 0.59162 | -0.75726 | 0.0084363 |
| 11 | ESI+ | 482.3193 | 14.73min | LysoPC(15:0/0:0) | HMDB0010381 | C23H48NO7P | 0.6895 | 0.84187 | -0.24832 | 0.043791 |
| 12 | ESI+ | 542.3217 | 11.93min | LysoPC(20:5(5Z,8Z,11Z,14Z,17Z)/0:0) | HMDB0010397 | C28H48NO7P | 0.6842 | 0.85128 | -0.23229 | 0.45349 |
| 13 | ESI+ | 1097.6746 | 11.82min | Ganglioside GM3 (d18:1/12:0) | HMDB0004842 | C53H96N2O21 | 0.7158 | 0.74152 | -0.43144 | 0.1306 |
| 14 | ESI+ | 247.1314 | 9.92min | 1α-1-Hydroxy-2,4(18),11(13)-eudesmatrien-12-oic acid | HMDB0037557 | C15H20O3 | 0.7053 | 1.0163 | 0.02329 | 0.89687 |
| 15 | ESI+ | 1086.4249 | 11.59min | 13Z,16Z-docosadienoyl-CoA | HMDB0062220 | C43H74N7O17P3S | 0.6912 | 0.58032 | -0.78507 | 0.0038834 |
| 16 | ESI+ | 986.2754 | 11.48min | trans-2-Enoyl-OPC4-CoA | HMDB0011120 | C35H54N7O18P3S | 0.6886 | 0.44345 | -1.1732 | 0.00020587 |
| 17 | ESI+ | 303.193 | 12.66min | 19-Oxotestosterone | HMDB0003959 | C19H26O3 | 0.7781 | 0.90453 | -0.14476 | 0.45524 |
| 18 | ESI+ | 289.2129 | 12.60min | Etiocholanedione | HMDB0003769 | C19H28O2 | 0.7246 | 0.97574 | -0.035437 | 0.9155 |
| 19 | ESI+ | 301.214 | 11.26min | 4-Oxoretinol | HMDB0012329 | C20H28O2 | 0.7202 | 1.0732 | 0.10186 | 0.7286 |
| 20 | ESI+ | 653.6428 | 22.35min | CE(18:0) | HMDB0010368 | C45H80O2 | 0.6939 | 0.87091 | -0.1994 | 0.81301 |
| 21 | ESI+ | 997.0251 | 22.68min | TG(18:1(11Z)/22:1(13Z)/22:1(13Z)) | HMDB0049349 | C65H120O6 | 0.7202 | 1.2011 | 0.26435 | 0.39708 |

**Table S2: Metabolites associated with clinical phenotype in patients with alcoholic liver disease with ascites**

| **Metabolite** | **Description** | **Increased or decreased in ascite** | **Exist in large ascites-associated fingerprint** |
| --- | --- | --- | --- |
| **Related to amino acids and proteolysis** | |  |  |
| L-Tryptophan | Tryptophan (Trp) or L-tryptophan is an alpha-amino acid. These are amino acids in which the amino group is attached to the carbon atom immediately adjacent to the carboxylate group (alpha carbon). | ↓ | ▲ |
| Allysine | Allysine (CAS: 1962-83-0), also known as 2-amino-6-oxohexanoic acid or 6-oxonorleucine, belongs to the class of organic compounds known as alpha-amino acids. | ↑ | ▲ |
| N6-Acetyl-L-lysine | N6-Acetyl-L-lysine is an acetylated amino acid. Post-translational lysine-acetylation is one of two major modifications of lysine residues in various proteins. The best-characterized mechanism is acetylation, catalyzed by histone acetyltransferase (HAT) enzymes. | ↓ | ▲ |
| Ne,Ne dimethyllysine | Ne,Ne dimethyllysine belongs to the class of organic compounds known as alpha amino acids. These are amino acids in which the amino group is attached to the carbon atom immediately adjacent to the carboxylate group | ↑ | ▲ |
| Benzoquinoneacetic acid | Benzoquinoneacetic acid (BQA) is an oxidized form of homogentisic acid. The oxidation of homogentisic acid to BQA is catalyzed by the enzyme polyphenol oxidase. | ↑ | ▲ |
| 5-Hydroxylysine | 2,6-Diamino-5-hydroxyhexanoic acid, also known as hydroxylysine or 5-hydroxy-2,6-diaminohexanoic acid, belongs to the class of organic compounds known as alpha amino acids. | **↓** | ▲ |
| **Related to fatty acid metabolism** | |  |  |
| (S)-3-Hydroxydodecanoyl-CoA | (S)-3-Hydroxydodecanoyl-CoA, also known as (S)-3-hydroxylauroyl-CoA, belongs to the class of organic compounds known as (s)-3-hydroxyacyl coas. | ↓ | ▲ |
| Oleic acid | Oleic acid (or 9Z)-Octadecenoic acid) is an unsaturated C-18 or an omega-9 fatty acid that is the most widely distributed and abundant fatty acid in nature. Oleic acid is used in manufacturing of surfactants, soaps, plasticizers. It is also used as an emulsifying agent in foods and pharmaceuticals. | ↑ | ▲ |
| (2E)-Decenoyl-CoA | (2E)-Decenoyl-CoA is a beta-oxidation intermediate, the substrate of the enzyme peroxisomal acyl-CoA thioesterase 2 (PTE-2, 3.1.2.2), which is localized in the peroxisome. | ↓ | ▲ |
| 1α-1-Hydroxy-2,4(18),11(13)-eudesmatrien-12-oic acid | 1α-1-Hydroxy-2,4(18),11(13)-eudesmatrien-12-oic acid belongs to the class of organic compounds known as sesquiterpenoids. These are terpenes with three consecutive isoprene units. | ↑ |  |
| 13Z,16Z-docosadienoyl-CoA | 13Z,16Z-docosadienoyl-CoA is classified as a member of the Very long-chain fatty acyl CoAs. Very long-chain fatty acyl CoAs are acyl CoAs where the group acylated to the coenzyme A moiety is a very long aliphatic chain of 22 carbon atoms or more.13Z,16Z-docosadienoyl-CoA is considered to be practically insoluble (in water) and acidic. | ↓ | ▲ |
| trans-2-Enoyl-OPC4-CoA | trans-2-Enoyl-OPC4-CoA belongs to the class of organic compounds known as 2-enoyl coas. These are organic compounds containing a coenzyme A substructure linked to a 2-enoyl chain. Thus, trans-2-enoyl-OPC4-CoA is considered to be a fatty ester lipid molecule. | ↓ | ▲ |
| (E)-8-Hydroxy-2-octene-4,6-diynoic acid | (E)-8-Hydroxy-2-octene-4,6-diynoic acid belongs to the class of organic compounds known as medium-chain fatty acids. These are fatty acids with an aliphatic tail that contains between 4 and 12 carbon atoms. | ↑ | ▲ |
| TG(18:1(11Z)/22:1(13Z)/22:1(13Z)) | TG(18:1(9Z)/20:1(11Z)/24:1(15Z)) is a mononervonic acid triglyceride. Triglycerides (TGs or TAGs) are also known as triacylglycerols or triacylglycerides, meaning that they are glycerides in which the glycerol is esterified with three fatty acid groups | ↑ | ▲ |
| **Related to phospholipid metabolism** | |  |  |
| LysoPC(15:0/0:0) | LysoPC(15:0) is a lysophospholipid (LyP). It is a monoglycerophospholipid in which a phosphorylcholine moiety occupies a glycerol substitution site. Lysophosphatidylcholines can have different combinations of fatty acids of varying lengths and saturation attached at the C-1 (sn-1) position. LysoPC(15:0), in particular, consists of one chain of pentadecanoic acid at the C-1 position. | ↓ | ▲ |
| LysoPC(20:5(5Z,8Z,11Z,14Z,17Z)/0:0) | LysoPC(20:5(5Z,8Z,11Z,14Z,17Z)) is a lysophospholipid (LyP). It is a monoglycerophospholipid in which a phosphorylcholine moiety occupies a glycerol substitution site. Lysophosphatidylcholines can have different combinations of fatty acids of varying lengths and saturation attached at the C-1 (sn-1) position. LysoPC(20:5(5Z,8Z,11Z,14Z,17Z)), in particular, consists of one chain of eicosapentaenoic acid at the C-1 position. | ↓ | ▲ |
| Ganglioside GM3 (d18:1/12:0) | Ganglioside GM3 (d18:1/12:0) is a glycosphingolipid (ceramide and oligosaccharide)or oligoglycosylceramide with one or more sialic acids (i.e. n-acetylneuraminic acid) linked on the sugar chain. It is a component the cell plasma membrane which modulates cell signal transduction events. | ↓ | ▲ |
| **Related to steroid and hormone** | |  |  |
| 19-Oxotestosterone | 19-Oxotestosterone is catalyzed by Aromatase (EC1.14.14.1), also called estrogen synthetase (a cytochrome P450 enzyme which catalyzes the formation of aromatic C18 estrogens from C19 androgens; it is symbolized CYP19) into oestrogens via sequential oxidations at the 19-methyl group. | ↓ | ▲ |
| Etiocholanedione | Etiocholanedione is a 5-beta metabolite product of the catabolism of androgens. Etiocholanedione has been identified as a ketosteroid and isolated from the urine of healthy and diseased persons. Etiocholanedione has been identified as a metabolite of an altered androgen metabolism that eventually leads hepatocellular carcinoma to impaired hormone responsiveness in human. | ↓ | ▲ |
| 4-Oxoretinol | 4-oxo-retinol, a metabolite of retinol synthesized in mouse embryonal carcinoma F9 cells, is active in inducing differentiation of these cells. It also functions as a ligand of retinoic acid receptors and a transcriptional activator of reporter. genes. 4-Oxoretinol is a metabolite of retinol in the human promyelocytic leukemia cell line NB4 which induces cell growth arrest and granulocytic differentiation | ↑ | ▲ |
| CE(18:0) | CE(18:0) is a cholesterol fatty acid ester or simply a cholesterol ester (CE). Cholesterol esters are cholesterol molecules with long-chain fatty acids linked to the hydroxyl group. They are much less polar than free cholesterol and appear to be the preferred form for transport in plasma and for storage. | **↓** | ▲ |
